# Enhancing the Impact Strength of Prepacked Aggregate Fibrous Concrete Using Asphalt-Coated Aggregates

**DOI:** 10.3390/ma15072598

**Published:** 2022-04-01

**Authors:** Nikolai Ivanovich Vatin, Gunasekaran Murali, Sallal R. Abid, Afonso Rangel Garcez de Azevedo, Bassam A. Tayeh, Saurav Dixit

**Affiliations:** 1 Peter the Great St. Petersburg Polytechnic University, 195251 Saint Petersburg, Russia; vatin@mail.ru (N.I.V.); sauravarambol@gmail.com (S.D.); 2 Civil Engineering Department, Wasit University, Kut 52003, Iraq; sallal@uowasit.edu.iq; 3 LECIV-Civil Engineering Laboratory, UENF-State University of the Northern Rio de Janeiro, Av. Alberto Lamego, 2000, Campos dos Goytacazes 28013-602, RJ, Brazil; afonso.garcez91@gmail.com; 4 Civil Engineering Department, Faculty of Engineering, Islamic University of Gaza, Gaza Strip P.O. Box 108, Palestine; btayeh@iugaza.edu.p

**Keywords:** impact strength, fibre, asphalt-coated aggregates, failure, ductility, prepacked aggregate concrete

## Abstract

The brittleness of plain concrete represents a significant issue to the integrity of concrete structures when subjected to impact loading. Recent rapid industrialization has attracted researchers to find a solution for concrete brittleness and enhance its ductility. In light of this, the prepacked aggregate fibrous concrete (PAFC) with single and double precoated coarse aggregates using asphalt is proposed and examined. Nine different mixtures were designed using polypropylene and steel fibre of 3% dosage with single and double asphalt-coated aggregates. Specimens were prepared with natural aggregate and 100% C-graded asphalt-coated aggregate to evaluate their impact strength. The ACI Committee 544 drop-weight impact standard was followed in the testing of all specimens. Results indicated that using asphalt-coated aggregate can improve the impact energies of concrete. The impact energy at cracking and failure of the single asphalt-coated aggregate specimen was 1.55 and 2.11 times higher, while the double-coated aggregate specimens exhibited 1.73 and 2.56 times greater than the natural aggregate specimen, respectively. The contribution of fibres in enhancing the impact resistance is remarkable compared to the single- and double-coated aggregates used in PAFC.

## 1. Introduction

The growing use of concrete materials has led to increasing concrete constructions being able to withstand frequent or infrequent impact loads [1]. Some typical examples of impact loading scenarios are: quake-prone structures in seismic zones, bomb detonation that damages the defensive structures, structures off the coast that are at risk of being hit by waves, the effect from airplanes landing on runways at airfields and industrial floors [2]. Plain concrete subjected to impact loading is restricted in its ability to deform and absorb energy, posing a significant challenge to their structural safety [3]. As a consequence, several energy-absorbing materials were added to improve the ordinary concrete resistance to impact, and significant development has been achieved in this area. Lu et al. [4] evaluated the energy-absorbent properties of concrete containing rigid and flexible particles. Their findings indicated that the adding of 20 and 40% flexible and rigid properties both enhanced energy-absorbent properties, with the former outperforming the other. Ma et al. [5] tested the energy-absorption capability of concrete containing coral aggregate and compared it with the performance of rock, sandstone and normal concrete. The maximum energy absorption ratio was found in coral aggregate concrete, suggesting the excellent resistance to impact. Li et al. [6] investigated self-compacting concrete comprising asphalt-coated aggregates and tested the mechanical and dynamic properties. A split Hopkinson pressure bar of 75 mm diameter was used to evaluate the dynamic properties of concrete. Results revealed that incorporating asphalt-coated aggregate into concrete enhances the impact toughness index and improves the resistance to impact. In addition, the concrete impact toughness index is highly dependent on the asphalt layer thickness that is looped around the surface of the aggregate. An asphalt layer thickness of 120 µm is ideal for this strain-rate range since the average improvement in impact toughness index is around 20%. Additionally, fibre materials are regarded as a great choice for improving the concrete impact resistance. The results of studies on different fibres revealed that when the strain rate rises, the fibres are pulled out or even separated, resulting in an important portion of the impact energy being absorbed by fibre breakage and its debonding from the concrete mixture [7,8,9]. As a result, concrete reinforced with fibres may consume far more impact energy than normal concrete when subjected to the same amount of harm, showing significantly better impact resistance [10,11].

Civil engineering technology has advanced at an alarming rate. Conventional fibrous concretes have evolved continuously with different forms; prepacked aggregate fibrous concrete (PAFC) is one of them. Placing coarse aggregate and fibre particles in an empty formwork and filling gaps with cement grout mixture is PAFC. Several investigations have been employed regarding the mechanical properties of PAFC and their impact-resistance performance. Mohammadhosseini et al. [12] examined the influence of polypropylene fibres on the mechanical characteristics of PAFC when subjected to increased temperatures of up to 600 °C. The results indicated that the mass-loss rate in PAFC mixtures containing polypropylene fibres was greater than in plain mixtures when exposed to high temperatures. The mass losses of 12 and 11% were observed for PAFC mixtures with 1% fibre at 600 °C under the gravity and pumping procedures, respectively. Alrshoudi et al. [13] reported 29.5% less shrinkage in the PAFC containing 0.75% polypropylene fibres compared to the control sample when the pumping method of grouting was used. Using a variety of steel fibre types and combinations, Murali and Ramprasad [14] studied the joint effect of layered and preplaced aggregate concrete slabs. The slab’s preparation and reinforcing were carried out in three layers using distinct fibres: crimped, hooked-end, and a hybrid combination. The layered slabs had a greater impact strength than a reference single-layer slab, even though they contained the same quantity of fibres. Abirami et al. [15] stated that PAFC specimens containing 5% of 5D hooked-end steel fibre resulted in improving the cracking and failure impact numbers by about 15 and 23 times higher than non-fibrous specimens. Abirami et al. [16] stated that the ductile behaviour that results from the increased impact energy absorption of multi-layered PAFC specimens was verified. However, none of the specimens lost their geometry shape or integrity of the structure after the impact. Manohar et al. [17] studied the composite fender, which is made up of corrugated steel plates on the inside and concrete panels on the outside. Compared to the PAFC panel, the preplaced aggregate concrete top non-fibrous panel was more vulnerable to central impact force. More ductility in the PAFC composite panel meant no further brittle failure. PAFC materials are a good option for the composite fender assembly’s exterior cover because of these advantages. In the recent past, there was a huge scope of producing higher impact-resistance material and fibrous concrete.

Based on the introduced literature, a material that can absorb energy and increase the impact resistance of PAFC in its hardened condition must be developed. In the present study, the concept of asphalt-coated aggregate with a single and double coating is used for producing the PAFC to evaluate their impact-resisting performance. Moreover, the combined effect of fibres (steel and polypropylene) and asphalt-coated aggregates were used to increase the impact energy absorption capacity of PAFC. The utilization of the single and double asphalt-coated aggregate in concrete is a new innovation in the technological development in the construction industry. This research area has still not been explored comprehensively and needs particular focus to implement this type of concrete in practice.

## 2. A Design Approach Based on the Theory of High-Speed Impact

**Figure 1:**
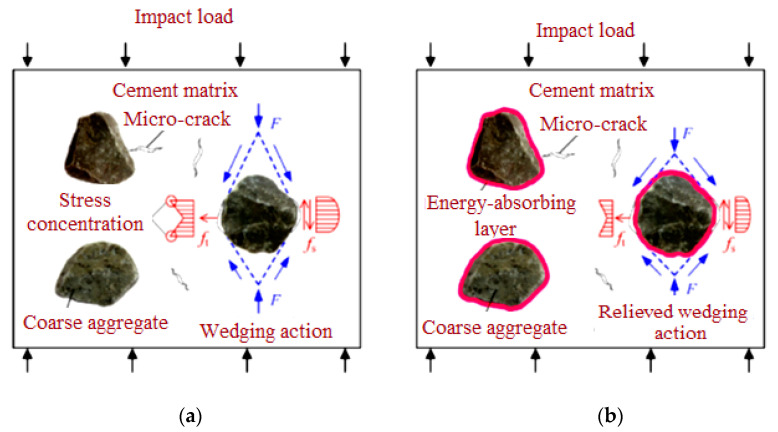
Schematic drawing of coarse aggregate failure (**a**) natural; (**b**) asphalt coated.

Impact loading is a form of dynamic loading that is distinguished by a rapid change in velocity that occurs in a short period. In line with the momentum theory, it is possible to considerably increase the impact load, followed by large kinetic energy. Structures often collapse due to their inability to absorb massive impact energy, such as in air transportation’s renowned bird strike catastrophes. Hence, impact resistance is thus determined by a structure’s combined energy absorption and load-bearing capabilities. The material’s load-bearing capability ensures structural stability and stiffness, whereas its energy absorption capability reduces the internal damage affected by a high impact. The impact load is sustained by the concrete matrix and aggregates in traditional cement-based concrete. Figure 1a graphically illustrates the stress concentration at the fracture point in the interface region due to the “wedging effect” caused by the differing elastic modulus of the aggregate and cement matrix. Microcracks develop, propagate, and link to disperse impact energy in traditional concrete’s minimal elastic deformation capacity [18]. As a consequence of the stress concentration in the interfacial region when exposed to impact loads, microcracks would rapidly form, resulting in the dramatic breakdown of structural concrete. As a result, optimizing the time-dependent micro-crack features is crucial for increasing the concrete’s impact resistance.

**Figure 2:**
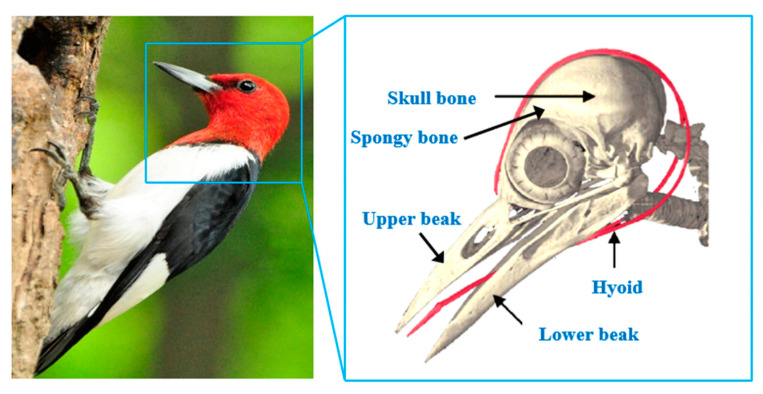
Woodpecker’s skull structure.

Inspiration can be drawn from nature’s bionic species to create PAFCs that are more resistant to impact. Biologically, the woodpecker is an outstanding demonstration of a predator endowed with ideal impact-resistance capabilities. Statistics show that a woodpecker may hammer a tree 12,000 times in one day at a maximum speed of 6–7 m/s without producing any brain damage or cerebral trauma [19]. The woodpecker’s distinctive skull anatomy is a major factor in the bird’s extraordinary ability to withstand impact. Typical woodpecker anatomy is shown in Figure 2. Several parts of the woodpecker’s body contribute to its ability to resist being drummed, but the tongue is particularly crucial. Figure 2 shows how the tongue stretches from the floor of the mouth to the back of the neck, where it detaches into two bands that surround and connect with the whole skull bone before coming forward to the nostril. The tongue structure is made up of a hyoid and a thin layer of muscle tissue outside the hyoid. While drumming a tree, the bone skeleton of its tongue acts as a load transmission mechanism, much like the aggregates in a cement matrix operate as load transmission mechanisms when the impact loads are applied to concrete structures under certain conditions. The soft muscle tissue layer absorbs a major percentage of the impact energy and transfers the remaining impact energy to other parts of the head rather than the brain. Thus, the brain is wholly shielded against serious shock damages in this manner. Because of their weak deformation capacity, aggregates in concrete constructions cannot release the impact energy. As previously described, asphalt may enhance the concrete’s ability to absorb energy. Hence, a woodpecker-tongue-like structure is created when the asphalt is applied over coarse aggregates, potentially improving concrete’s resistance to impact.

The coarse aggregate covered with asphalt is seen in Figure 1b. Coarse aggregates’ “wedging effect” is alleviated by using a flexible energy-absorbing layer, which improves crack propagation’s time-dependent properties by reducing stress concentration at interfaces. Under static loads, a previous study has shown that asphalt cohesiveness is the most common form of failure for asphalt and cement [20]. Reclaiming the asphalt-integrated concrete’s energy absorption, toughness, and fracture energy have been significantly increased due to asphalt’s crack-arresting and energy dissipation capabilities [21]. Several tests were carried out, described in the following section, to validate the mechanical properties and impact resistance of PAFC.

## 3. Experimental Work

### 3.1. Materials

**Figure 3:**
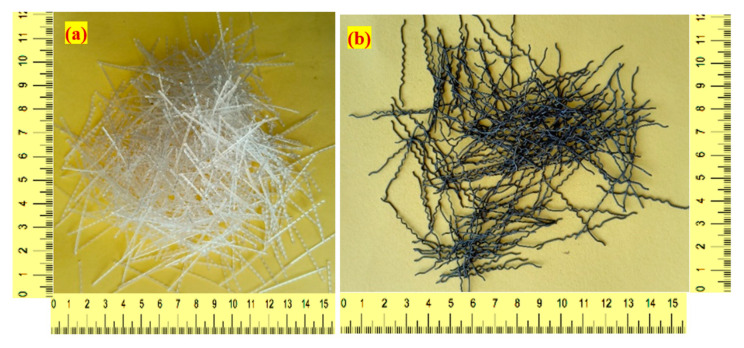
Shape of fibres (**a**) PF (**b**) SF.

**Figure 4:**
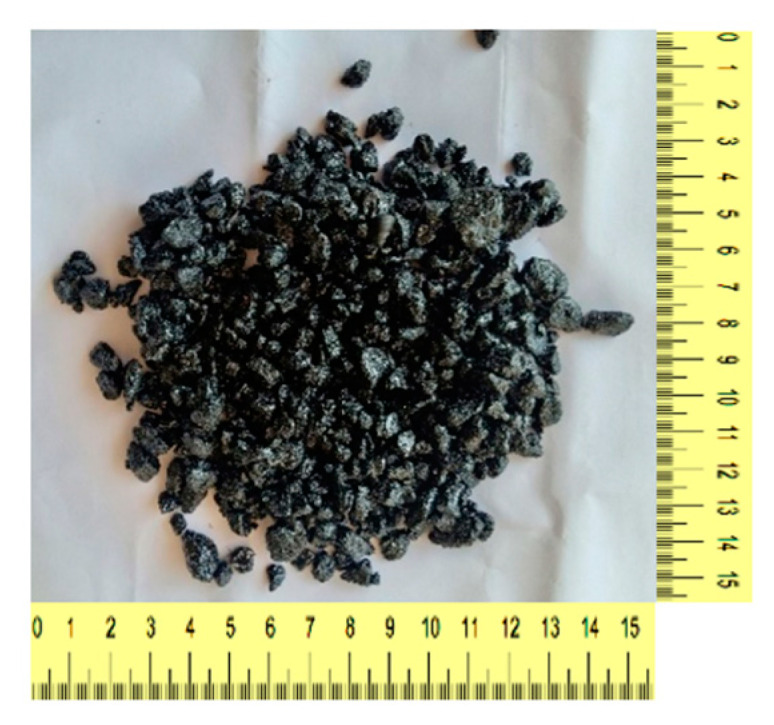
Asphalt-coated aggregates.

According to IS: 1489-2015 [22] specification, a pozzolana Portland general-purpose cement was used in this investigation. The specific gravity of cement was 3.09 kg/m^2^ and its specific surface area was 318 kg/m^2^. River sand possessing a fineness modulus of 2.39 and water absorption of 1.14 was utilized as a fine aggregate in the final product. In compliance with ASTM C939 [23], it had a particle size of not more than 2.36 mm. Consequently, a flowable grout was developed to fill up the gaps. The maximum size of the used gravel (coarse aggregate) was 12.5 mm with water absorption of 0.59% and a specific gravity of 2.6%. A high-range water reducer (Tec Mix 640) was used to produce the flowable grout, where different doses (0.3 to 0.5% by cement weight) were utilized to generate the flowable grout for non-fibrous and fibrous specimens, respectively. A 50 mm-long and 1.0 mm diameter hybrid hooked end-crimped steel fibre (SF) with a tensile strength of 1200 MPa was used in three fibrous mixtures, while in another three similar mixtures, polypropylene fibre (PF) with 500 MPa tensile strength, 45 mm length and 0.8 mm diameter was used. Figure 3 illustrates the shape of the SF and PF employed in this study. In this study, VG 40 asphalt was employed with the reported physical properties listed in Table 1. Two asphalt thicknesses of 50 and 100 µm were used in the single and double coatings, respectively. Figure 4 depicts the surface of coarse aggregate precoated with asphalt.

### 3.2. Process of Applying Asphalt Coating to Aggregates

**Figure 5:**
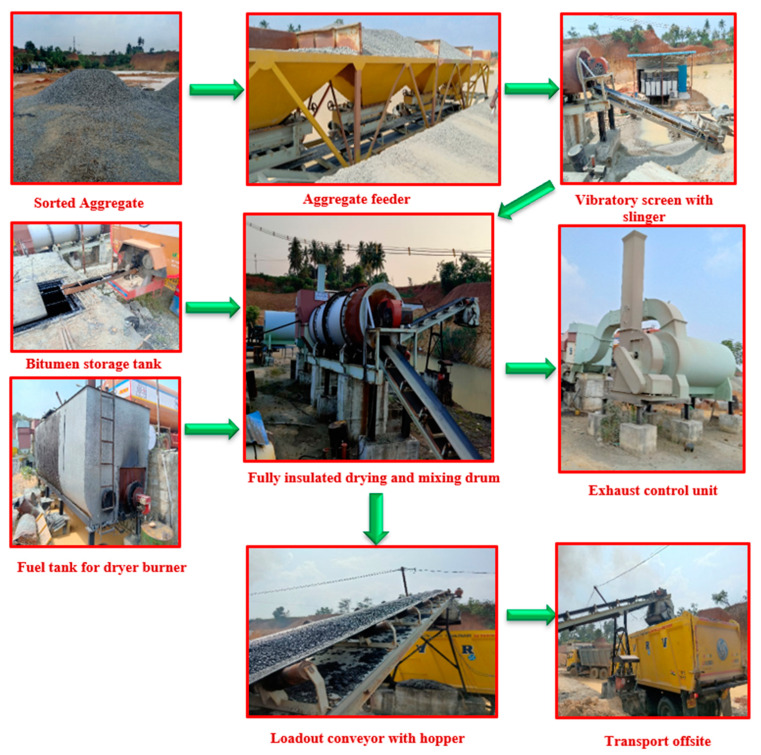
Preparation and production of coarse aggregate coated with asphalt.

Figure 5 depicts the procedure of applying asphalt to aggregates in a sequential manner. Three aggregate feeders were used to feed specified quantities and sizes of stockpiled aggregates into the mixing container. The big aggregates were removed from the mix and kept out of the mixing drum using a vibrating screen. A conveyor belt transported them all to the mixing drum at once. After the vibratory screen, the aggregates were delivered to the drying container. The dryer maintains a high temperature that allows for the removal of moisture from the aggregates. The burner unit is critical in this process, and the elevating system of the drying drum flights ensures that the aggregate heats up uniformly throughout the drying drum flights. Following this procedure, the required amount of asphalt in liquid form is pumped into the drum and allowed to revolve at various speeds. Using a drying burner, asphalt was extracted from the vehicle and kept in a tank before being transferred to the mixing drum. The mixing drum’s asphalt-coated aggregate was transported to a loading conveyor for loading into the vehicle. Dry dust and hazardous smoke were caught in the discharge control unit before being discharged into the atmosphere during this operation. A set of switches in the cabin controls the whole input/output operation.

### 3.3. Mix Combinations

**Figure 6:**
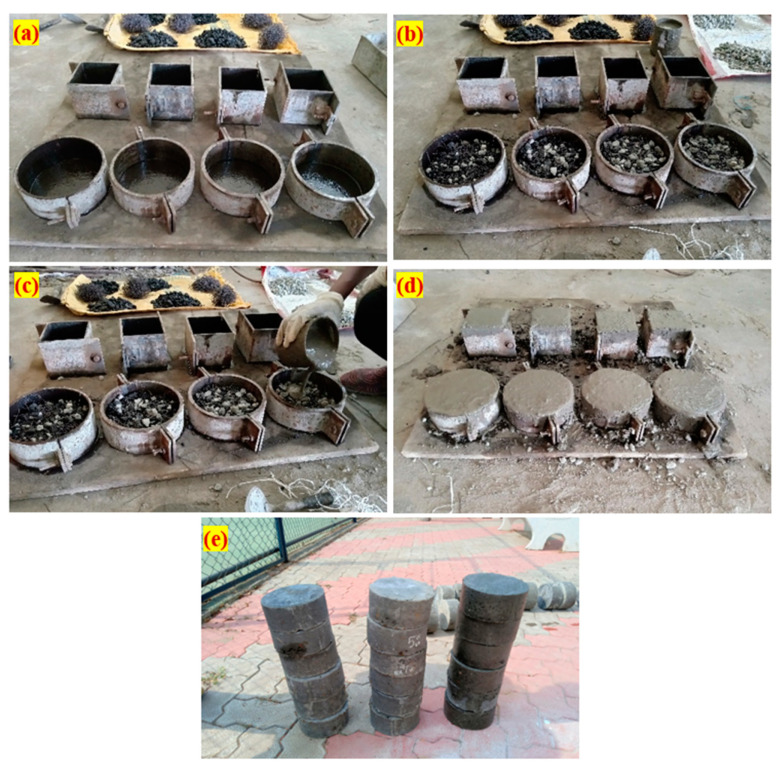
PAFC casting procedure (**a**) empty mold, (**b**) fibres and aggregates are filled in the mold, (**c**) grouting under gravity, (**d**) finished specimens and (**e**) specimens after demolding.

With an optimal sand/binder (s/b) ratio (1.0) and binder/water ratio (0.45), nine different concrete mixes were made and evaluated in this research. A set of flow cone experiments performed in accordance with ASTM C939 [23] determined these ratios to be appropriate to meet an efflux time of 35–40 ± 2 s. These ratios and a slight compaction during grouting eliminated honeycombing in the concrete after casting to the full extent. The mixing combination of nine mixtures with and without fibres and single and double asphalt coatings of aggregates are shown in Figure 6. The first mixture was named NC, comprising 100% natural aggregate without fibres. The second and third mixtures were named NC-C1 and NC-C2, respectively, indicating a single and double coating without fibres. The C1 and C2 from the mixtures denote the single and double asphalt coatings, respectively. The next three mixtures were reinforced with PF and the exact composition of the first three mixtures was used. These mixtures were named P-PF, P-PF-C1 and P-PF-C2. The last three mixtures were reinforced with SF and the mixing combination pattern is the same as the PF-based mixtures. These series were named P-SF, P-SF-C1 and P-SF-C2. The mixing composition of nine mixtures were demonstrated in Table 2.

### 3.4. Specimen Preparation Procedure

Using cylindrical specimens with dimensions of 152 mm in diameter and 64 mm in height, the impact strength of the PAFC was tested. The compressive strength was assessed using the 100 mm cubical specimens. For each combination, three specimens were made, and the mean value was given and discussed. The stage-by-stage method of PAFC casting, as shown in Figure 6, comprised three important steps. Before filling the cylindrical mould, a lubricant was applied to all of the inner side faces, as illustrated in Figure 6a. Second, as Figure 6b illustrates, a natural skeleton was produced by merging pre-mixed fibre and aggregates. Cement grout was then poured as a final stage and was permitted to fill the gaps by gravity. As indicated in Figure 6c, the grout was gently compacted to ensure that all gaps were sealed. This kind of compaction eliminated gaps and prevented honeycombing. Figure 6d depicts the appearance of grouted specimens. After 24 h, all specimens were demoulded, and the appearance of sampled specimens was grouped in Figure 6e. Before testing, all specimens were cured using a 28-day water immersion cure.

### 3.5. Impact Test Device and Methodology

**Figure 7:**
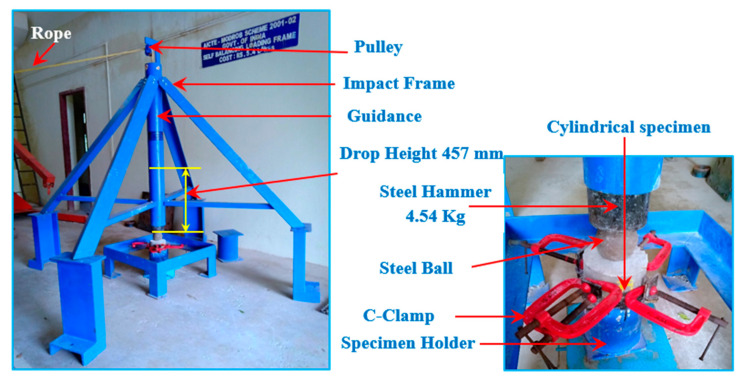
Impact test setup.

The drop-weight impact test was used to assess the PAFC specimens’ impact strength following the recommendations of ACI Committee 544 [30]. This study used the drop-weight impact testing system shown in Figure 7. There was no need for supplementary measurements for vibration, strain, or time history while using the drop-weight impact method. In this experiment, a steel hammer of 4.45 kg mass free-fell from a 457 mm height and was permitted to drop onto the specimen’s top surface in the same position. Four positioning legs limited the specimen’s horizontal mobility during testing. Two numbers O1 and O2 must be recorded for each specimen, which are the numbers of impact blows required to initiate specimen cracking (O1) and to fail it (O2). Failure was defined as a fracture that reached the bottom of the specimen and broke it in half. Equation (1) was used to compute the delivered impact energy.

(1) Impact energy=n×m×g×h

where *g*: gravity acceleration, *h*: hammer drop height, *m*: mass of hammer, and *n*: impact numbers.

This study is focused on the impact resistance of fibrous concrete by combining two different concepts. First, the preplaced aggregate fibrous concrete is produced with two different fibres and the gravity method of grouting was applied. Second, the natural aggregates were precoated with asphalt in a single and double layer before being used in concrete production. The combined effect of fibres and asphalt-coated aggregate on the impact resistance has been studied extensively. This research has studied the compressive strength, number of impact blows required to initiate specimen cracking and to fail it, ductility index, failure pattern, and failure mechanism.

## 4. Discussion of Results

### 4.1. Influence of Asphalt Coating on Compressive Strength

**Figure 8:**
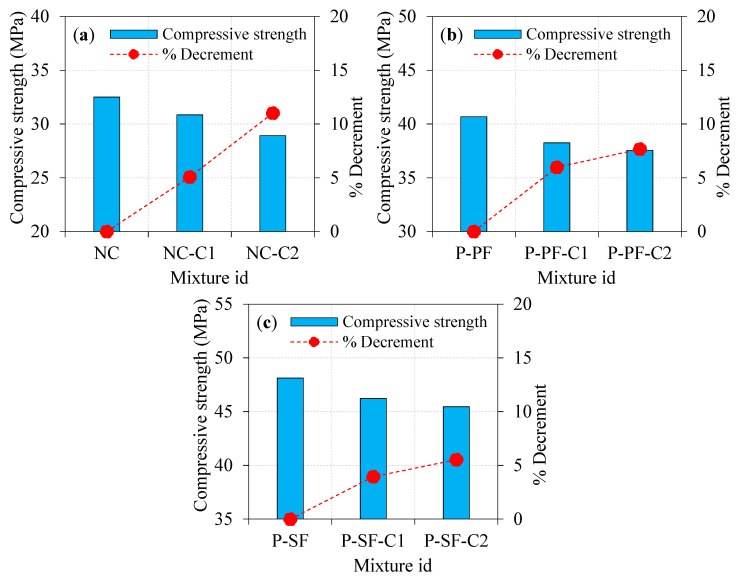
Effect of asphalt coating on compressive strength (**a**) plain specimens (**b**) PF-based specimens. (**c**) SF-based specimens.

**Figure 9:**
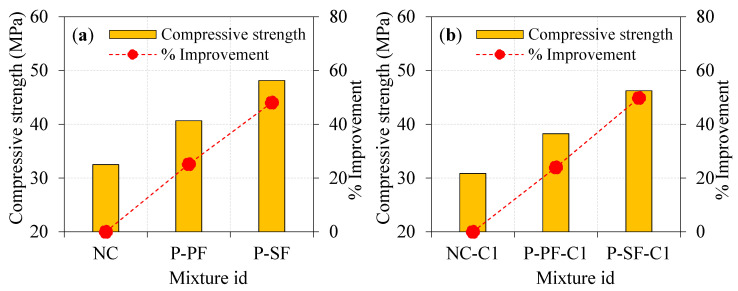

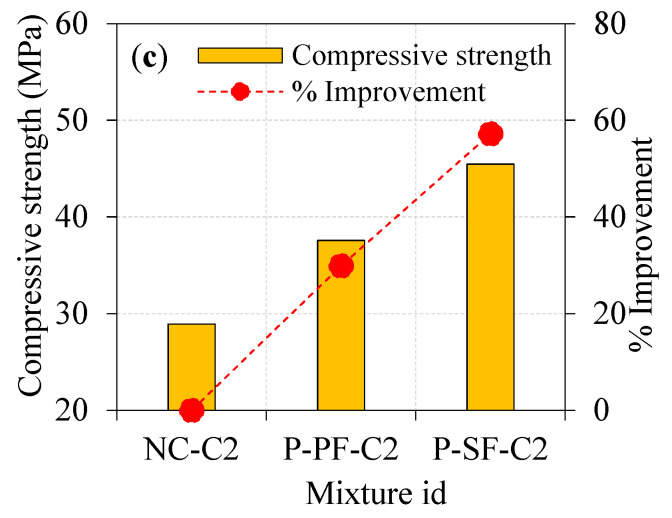
Effect of fibre type on compressive strength: (**a**) natural aggregate, (**b**) singly coated aggregate, (**c**) doubly coated aggregate.

The compressive strength results attained from the laboratory test for all specimens are presented in Figure 8 and Figure 9. It can be viewed prudently from Figure 8a that the specimen with single and double asphalt-coated aggregates exhibited a lower compressive strength compared to the natural aggregate’s specimens. For instance, the NC-C1 and NC-C2 specimens showed compressive strength reductions of about 5.08 and 11.02%, respectively, compared to the NC specimen. The reduced compressive strength may be due to the poor connection between the asphalt-covered aggregates and the concrete matrix. According to Debbarma et al. [31]’s study, when the high-aged recycled asphalt pavement was used, the compressive strength might drop by around 9 to 37%. Typically, plain concrete’s breakdown is accompanied by coarse aggregate fracturing because of its strong connection with the cementitious matrix. For the concrete comprising a single asphalt-coated aggregate, the bonding between the aggregate and the cementitious matrix was weak to a certain extent. Other studies have reported comparable results [32], which found that cement and asphalt cohesiveness was the primary failure cause [33]. The research showed a drop in compressive strength with increased asphalt coating from single to double.

While asphalt-coated aggregates had a moderate effect on compressive strength, PF inclusion increased it to a higher amount, as shown in Figure 8b. Compared to the P-PF specimens, the reduction in compressive strength was observed by 5.97 and 7.67% for the P-PF-C1 and P-PF-C2 specimens, respectively. The combined effect of the asphalt-coated aggregate and PF exhibited a lower reduction in compressive strength, particularly for the case of double-coated aggregates. However, the influence of the single-coated aggregate in PF-based concrete was less compared to double-coated aggregate. Decreasing compressive strength is approximately similar to the trend observed in non-fibrous concrete. Adding a second coating layer to the aggregate resulted in poor bonding, which resulted in an additional decrease in compression strength. Aggregates with excellent interlocking led to increased strength, but this effect could not be seen in either single- or double-coated aggregates. The compressive strength reduction in specimens comprising double-coated aggregates was greatly enhanced by adding PF. PF’s role in bridging microcracks formed in P-PF-C2 specimens prevented a greater decrease in compressive strength when double-coated aggregate was used.

Asphalt-coated aggregate and SF’s combined action on compressive strength is remarkable. As displayed in Figure 8c, the compressive strength reduction in P-SF-C1 and P-SF-C2 specimens was 3.95 and 5.53%, respectively, compared to the P-SF specimen. The poor bonding between the concrete matrix and asphalt-coated aggregate caused intrinsic flaws in the concrete mixture, resulting in lower compressive strength. For the aggregate with a double asphalt coating, the fracture would penetrate through the asphalt layer more readily, reducing the compressive strength. Brand and Roesler [33] stated that for asphalt-based concrete mixtures, asphalt cohesion failure was more significant than the asphalt–cement adhesion failure. The failure due to asphalt cohesiveness can be minimized by adding the SF into the concrete. SF has a greater impact on compressive strength than asphalt-coated aggregate does. SF and asphalt-coated aggregate’s combined action showed higher compressive strength and lessened the reduction in strength due to the increase in the coating from single to double [34].

### 4.2. Influence of Fibre Type on Compressive Strength

The effect of SF and PF on the compressive strength of PAFC comprising single- and double-coated aggregate is illustrated in Figure 9. Adding fibres can greatly enhance the compressive strength of concrete irrespective of the asphalt-coated aggregate used. For instance, P-PF and P-SF specimens comprising 100% natural aggregate showed a notable improvement in compressive strength by 25.17 and 48.06%, respectively, compared to NC. Likewise, the P-PF-C1 and P-SF-C1 specimens had 23.99 and 49.82% enhancements in compressive strength, respectively, compared to NC-C1. The maximum compressive strength was obtained from the SF-based specimens, which improved the strength by 29.88 and 57.19% for the P-PF-C2 and P-SF-C2 specimens, respectively, due to the presence of SF, which has a remarkable capacity to bridge micro/macro cracks. Consequently, more energy is needed to pull out and fibre debond as the fracture channel becomes tortuous [9,35]. In a nutshell, SF had a greater influence on concrete’s compressive strength than PF. This phenomenon is due to the tensile strength of the PF being significantly lower compared to the SF. It is usual practice to restrict the amount of fibre in a composite to 2% because of concerns about workability and the dispersion of fibres and the formation of fibre balls and voids, which reduces compressive strength [36,37]. The PAFC technique, on the other hand, eliminates this issue by pre-packing the fibres and coarse particles before pouring the grout. Remarkably, PAFC specimens had greater compressive strength due to the used 3% dosage of fibres. Consequently, a superior compressive strength was achieved due to the better resistance to crack initiation and propagation [38].

### 4.3. Impact Test Results

Table 3 summarizes the mean values of the impact blows required to initiate specimen cracking (O1) and fail it (O2) and the calculated impact energies of the tested specimens.

#### 4.3.1. Effect of Asphalt Coating on Impact Energies

**Figure 10:**
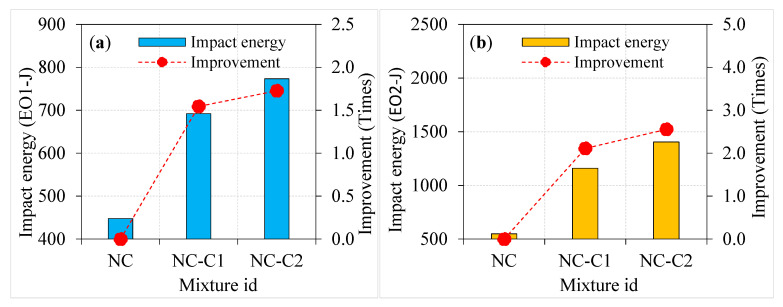
Effect of asphalt coating on impact strength of non-fibrous specimens (**a**) EO1 and (**b**) EO2.

The impact energy of the specimens was improved when 100% asphalt-coated aggregate was used instead of natural aggregate. Moreover, increasing the asphalt coating from single to double layer resulted in a sharp rise in impact energy at cracking and failure, as shown in Figure 10. The energy records at cracking (EO1) and failure (EO2) of NC were 447.8 and 549.5 J, respectively. As a consequence of this finding, it was determined that the specimen failed suddenly after breaking, resulting in a brittle fracture. As can be seen in Figure 10a,b, adding more layers of asphalt coating to the aggregate from single to double led to an increase in the impact energies (EO1 and EO2). For instance, the single-coated aggregate specimen (NC-C1) exhibited EO1 and EO2 values of 1.55 and 2.11 times higher, respectively, compared to the NC specimens. Likewise, the EO1 and EO2 values were 1.73 and 2.56 times higher for the NC-C2 specimen. Findings revealed that the specimens containing double-coated aggregates displayed exceptional impact resistance on par with specimens containing single-coated aggregates and 100% natural aggregate. The “wedging effect” of the aggregate in the cementitious matrix under impact loads was alleviated by a double asphalt coating, which served as cushioning. As a result, crack tip stress concentration surrounding the aggregates was effectively reduced, resulting in a slowdown in crack growth. Consequently, multiple cracks were produced, which dispersed more energy and increased the impact resistance of the specimen. As impact numbers increased, the specimens deteriorated and broke into two pieces; this contributed to their failure. Similar findings were observed in the earlier literature [34] and are confirmed in the observed failure pattern discussed in Section 4.3.4. An excellent post-crack resistance was seen in the specimens when single- and double-layer asphalt-coated aggregates were employed.

**Figure 11:**
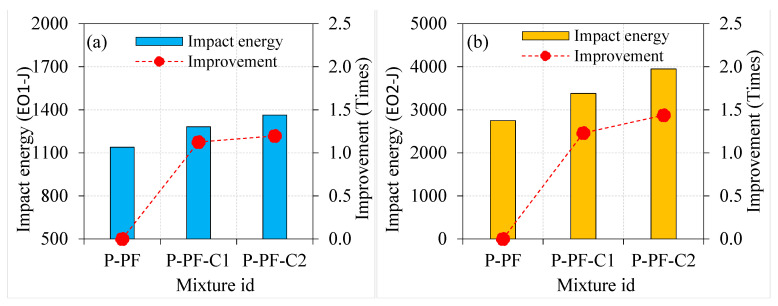
Effect of asphalt coating on impact strength of PF-based specimens (**a**) EO1 and (**b**) EO2.

Generally, adding fibres into PAFC results in an inherent improvement in impact energies. The impact energies at cracking and failure for the P-PF specimens were 1139.8 and 2747.7 J, respectively. The addition of PF in PAFC comprising single- and double-coated aggregates displayed a positive effect, as shown in Figure 11. For instance, the EO1 and EO2 values of the P-PF-C1 specimen were improved by 1.13 and 1.23 times, respectively, compared to the P-PF specimens. For P-PF-C2 specimens, an improvement was observed, 1.20 and 1.44 times higher for the EO1 and EO2, respectively. Although the incorporation of asphalt-coated aggregate had a significant influence on impact strength, the effect of PF remains noteworthy. In PAFC, the cumulative impact of PF and asphalt-coated aggregate demonstrated exceptional performance against impact load, which is attributed to the asphalt’s viscoelastic nature, which enveloped the coarse aggregates and prevented them from disintegrating [39]. At the same time, natural aggregates tend to break when subjected to impact loads. The bonding between the coarse aggregate and cement matrix is decreased due to the repeated impact load, which causes breaks in the natural aggregate. However, PF bonded with coated aggregates more strongly and alleviated the possibility of disintegration, resulting in higher impact energy absorption ability [34].

**Figure 12:**
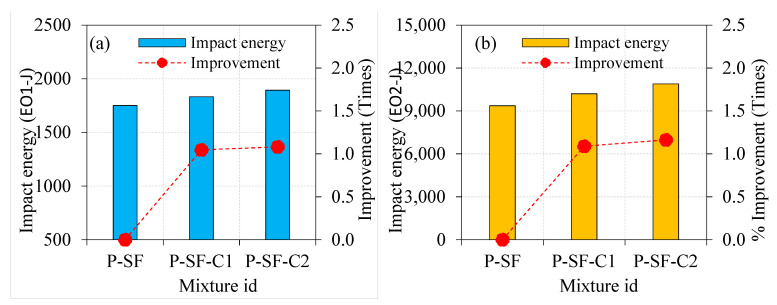
Effect of asphalt coating on impact strength of SF-based specimens (**a**) EO1 and (**b**) EO2.

Figure 12 depicts the influence of asphalt coating to the aggregate surface on the impact energies of SF-based PAFC. The highest impact energy absorption of PAFC was observed in this series of specimens. Compared to PAFC specimens comprising single-coated aggregates, the impact energy absorption of double-coated aggregate-based PAFC was superior in the case of SF inclusion. The EO1 of P-SF-C1 and P-SF-C2 specimens improved by 1.05 and 1.08 times compared to the P-SF specimen, as shown in Figure 12. At the same time, the EO2 of P-SF-C1 and P-SF-C2 specimens improved by 1.09 and 1.16 times. It is clear that increasing the asphalt coating from single to double resulted in a marginal enhancement in cracking and impact energies with the SF combination. Although that the maximum impact energies were observed in this series, the effect of asphalt coating was inferior compared to the steel fibre. However, SF and asphalt-coated aggregate’s combined action displayed favourable results regarding impact energies. Li et al. [6] reported that when the thickness of the asphalt coating exceeded 120 µm, the impact toughness of the composite decreased significantly. The impact resistance of the asphalt-coating-based PAFC was not reduced in our investigation because the asphalt coating thickness was employed under 120 µm for both single and double coatings. Furthermore, the viscoelastic character of asphalt caused PAFC to be more deformable with higher impact energy absorption. Moaveni et al. [40] stated that a higher coating thickness asphalt on the surface of the aggregate displayed a significant strength decrease. Cracks may pass freely through the asphalt layer when there is a greater concentration of asphalt coating on the aggregates [41], which reduces impact resistance. In this research, aggregate covered with a thinner layer of asphalt coating showed enhanced impact resistance.

#### 4.3.2. Effect of Fibre Type on Impact Energies

**Figure 13:**
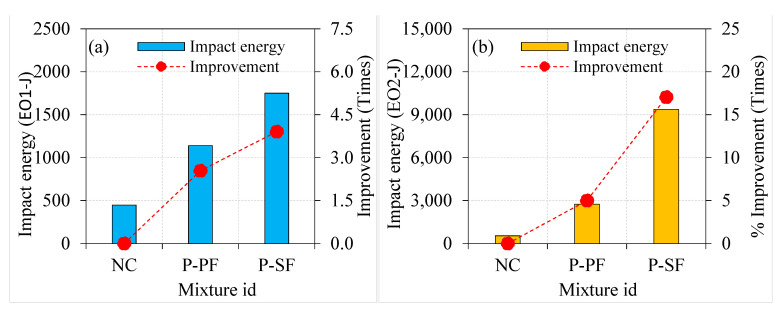
Effect of fibres on natural aggregate specimens (**a**) EO1 and (**b**) EO2.

**Figure 14:**
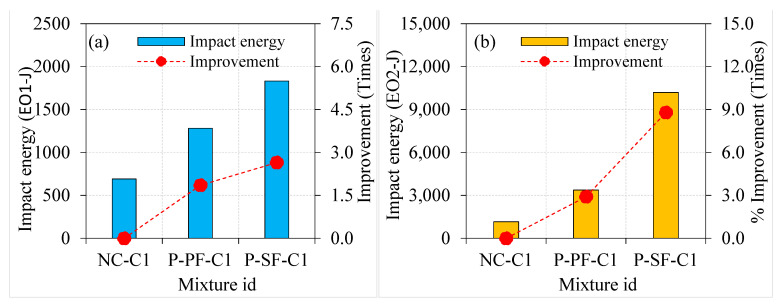
Effect of fibres on single-coated aggregate specimens (**a**) EO1 and (**b**) EO2.

**Figure 15:**
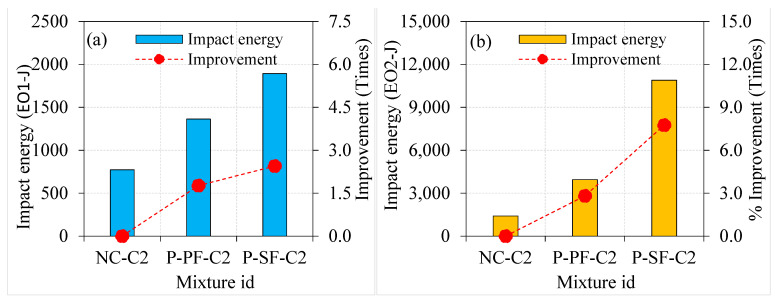
Effect of fibres on double-coated aggregate specimens (**a**) EO1 and (**b**) EO2.

Figure 13, Figure 14 and Figure 15 depict the influence of PF and SF on the impact energies of PAFC comprising single and double asphalt-coated aggregates. Both PF and SF addition to concrete can potentially increase the cracking and failure impact energies. As depicted in Figure 13, the EO1 and EO2 values for the P-PF specimens were 2.5 and 5.0 times higher compared to the NC specimen. At the same time, these values were 3.9 and 17.4 times higher for the P-SF than NC specimens. The fibre-reinforcing mechanism in the concrete matrix against impact stresses is solely responsible for this phenomenon [42]. The bridging action of solitary fibres is the most important attribute of these fibres. The fibres begin their true action with the micro-cracks’ commencement prior to the first cracking surface [43]. Consequently, the cracking impact resistance is increased by the dispersed fibre. The fibres crossing the two sides of the crack are subjected to increased tensile stresses after the initial surface crack forms because of the repeated impacts [44]. Consequently, the fibres become perfectly effective as the reinforcing elements that endure the tensile stresses delivered across the cracks and prevent the specimen from cracking and breaking [45]. This action continues up to the point at which the applied stresses may weaken each fibre link with its concrete matrix and the entire functional bridging action. This happens when the bridging tensile stress surpasses the fibre’s tensile strength [11]. As a result, fibre activity in crack bridging following crack development is substantially greater than fibre action before the emergence of the first cracking, which clarifies the reason for EO2 values and corresponding enhancement over EO1 values of specimens as can be seen in Figure 13, Figure 14 and Figure 15.

The impact energies corresponding to cracking and failure showed prominent enhancements, for the single and double asphalt-coated aggregate specimens as displayed in Figure 14 and Figure 15. Compared to NC-C1, the EO1 and EO2 values were 1.85 and 2.91 times higher for the P-PF-C1 specimens. Likewise, these values were higher by 2.65 and 8.79 times for the P-SF-C1 specimens. Because SF has greater bridging properties than PF, this finding may be ascribed to their superior performance in this application. Another note: the tensile strength of SF is greater and creates better bonding character with the concrete matrix, which accounts for this [35]. The combined action of double-coated aggregate with fibres showed an affirmative outcome on the impact energies. For instance, the P-PF-C2 specimens exhibited 1.76- and 2.81-times higher values in EO1 and EO2, respectively, as compared to NC-C2. Compared to the PF and asphalt-coated-aggregate-based PAFC, SF-based PAFC had a far greater influence on impact energies. The EO1 and EO2 recorded for the P-SF-C2 specimen were 2.45 and 7.75 times higher as compared to NC-C2, respectively. A considerable impact energy enhancement was noticed in PAFC when SF and asphalt-coated aggregate were mixed, owing to SF’s high tensile strength. The SF and PF demonstrated the capacity to increase the tensile strength and cohesion, resulting in distinguished physical improvements to the asphalt-coated aggregate fibrous concrete [46]. Fitzgerald [47] found that the asphalt-based concrete with fibre incorporation led to enhancing the strength, fatigue behaviour and ductility owing to the fibres’ inherent interaction with asphalt. Fibres act as a fracture shield and are capable of withstanding high tensile stresses and prevent crack development and propagation [48].

#### 4.3.3. Ductility Index of PAFC

**Figure 16:**
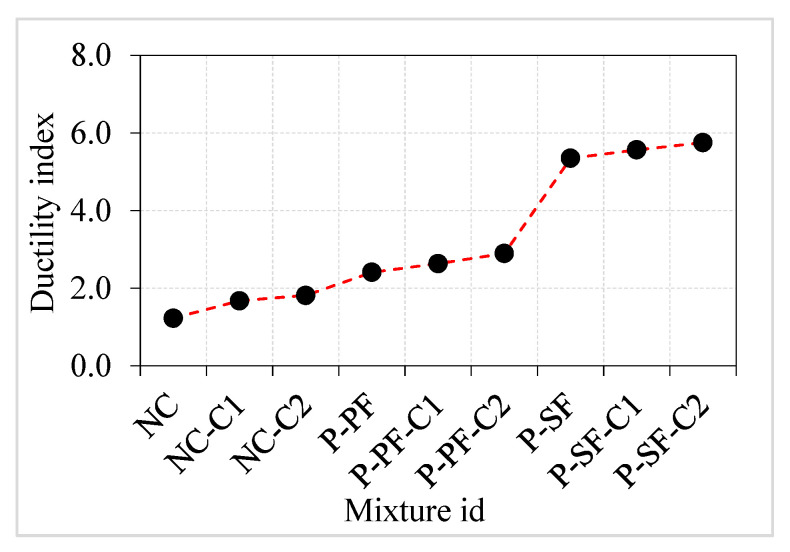
Ductility index.

Flexural ductility refers to the capacity of flexural members such as beams and slabs to survive plastic deformation. The ductility index is derived by dividing the failure deflection by the yielding point deflection [49]. Elasticity and plasticity are expected to exist in this formulation’s relationship between deformation and load. The cracking impact energy may be assumed to be the end of elastic behaviour and the beginning of plastic behaviour for the disc specimens. A simple definition of impact ductility index (ID) may be expressed as the ratio of the failure impact energy to the cracking impact energy. In many previous studies, this concept was used to discriminate between the impacts of various fibre materials when subjected to multiple impact loads [50,51]. The calculated impact ductility index of all PAFC specimens is shown in Figure 16. The ductility index value of NC, NC-C1 and NC-C2 specimens were 1.2, 1.7 and 1.8, respectively. The lowest values of ductility index indicated a lower post-cracking resistance, resulting in sudden failure after few numbers of impact blows. Moreover, using single- and double-coated aggregates in concrete displayed a marginal improvement in the ductility value, which also indicated a sudden failure.

The ductility index value of PAFC was improved by adding PF and coated aggregate. For instance, the recorded ductility value of P-PF, P-PF-C1 and P-PF-C2 specimens was 2.4, 2.6 and 2.9. Enhanced post-cracking behaviour of the PF specimens is attributable to the bridging effect of the PF fibres, which postponed breakdown and raised variations between cracking and failure impact energies. A remarkable enhancement in the ductility index was observed in the SF-based specimens. The P-SF, P-SF-C1 and P-SF-C2 specimens exhibited ductility index values of 5.3, 5.6 and 5.8, respectively. Higher failure impact energies resulted from SF’s excellent ductility, which is linked to its higher tensile strength and superior configuration when compared to PF. It is clear from the above discussions that the influence of fibres on ductility index was considerably higher, while asphalt-coated aggregate displayed marginal positive results.

#### 4.3.4. Failure Mode of TSFC

**Figure 17:**
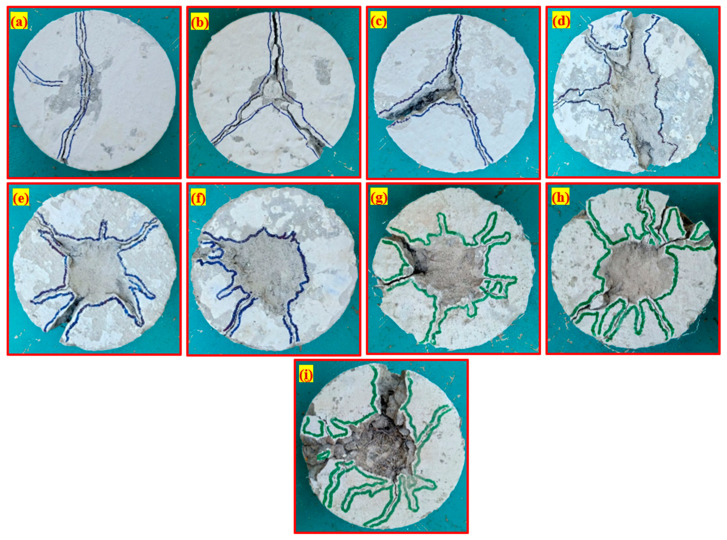
Failure modes of PAFC (**a**) NC, (**b**) NC-C1, (**c**) NC-C2, (**d**) P-PF, (**e**) P-PF-C1, (**f**) P-PF-C2, (**g**) P-SF, (**h**) P-SF-C1 and (**i**) P-SF-C2.

**Figure 18:**
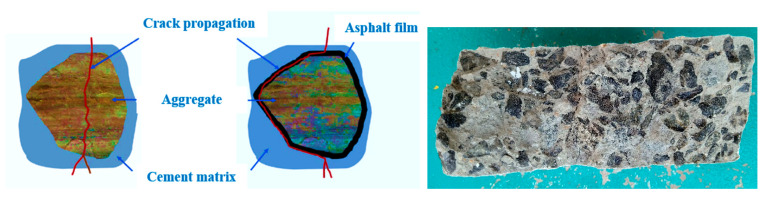
Aggregate failure mechanism.

Figure 17 shows the failure modes of all examined specimens when exposed to impact load. Figure 17a–c indicate brittle failure in the NC, NC-C1, and NC-C2 specimens after only a few impact blows (a–c). Asphalt-coated aggregate added to concrete did not affect the brittle failure mode. Non-fibrous cylindrical specimens were commonly subjected to this form of brittle failure, as reported in the scientific literature [10,52]. As a result of concrete’s heterogeneity and brittleness and the absence of any bridging elements, this behaviour was observed. In contrast, as seen in Figure 17d–i, all PAFC specimens showed many micro-cracks on the impact face that propagated outward from the impact point to the cylindrical specimen’s ends. As a result of adding fibres to PAFC, the cracking structure on the specimen’s top surface may be transformed from a single main crack to numerous cracks. Because of the fibres’ ability to prevent fracture development, the PAFC specimens did not collapse into numerous large fragments. Concrete pieces could not be expelled due to the fibre bridging effect. Fibres changed their breaking modes from brittle to ductile, resulting in less damage of the fracture. At the same time, asphalt-coated aggregates performed a supportive role in improving the ductility of PAFC. In terms of disaster preparation, it is important to note that the ductile breakdown mode extends evacuation timeframes. Results from this study were in line with earlier studies that reported the same failure mode [34,53]. In general, the failure happens in the natural aggregate-based concrete through the aggregates due to the excellent bonding between the mortar and aggregate (Figure 18). Rocco and Elices [54] reported that cracks might also occur around the round aggregates based on the mortar’s binding character to the aggregate. For PAFC, the low cohesiveness of the asphalt layer surrounding the aggregate stopped this crack from growing, as shown in Figure 18.

#### 4.3.5. Mechanism of PAFC Failure

**Figure 19:**
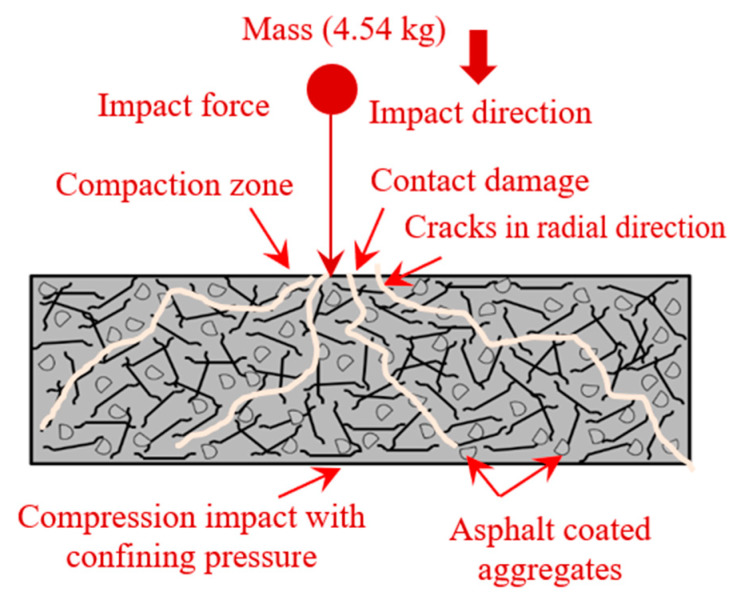
Mechanism of failure.

As a result of the impact loading, four distinct defects were observed in PAFC. First, repetitive impact force was delivered to the cylindrical specimen, resulting in localized contact destruction [37]. Secondly, owing to the generated shear stress and shear strain in a transverse direction, internal delamination of the matrix happened [55]. Another factor contributing to cement matrix failure was the drop mass impact that generated tension and compressive waves [15]. Finally, debonding failure of fibres from the cement matrix caused substantial damage to the strength and integrity of concrete, and it was tough to detect while in operation [37]. As previously stated, the time duration for such failures was condensed, making it impossible to identify the actual sequence of events that occurred [38]. The PAFC failure mechanism subjected to repeated impact is shown in Figure 19. The figure shows the failure mechanism for the SF-based specimens. However, the failure mechanism for PF-based concrete is the same as SF-based concrete. Both fibres played the same role in bridging cracks and improving impact resistance. The contribution of SF is significantly higher than the PF due to the higher tensile strength of fibres.

## 5. Conclusions

Asphalt-coated aggregates and fibres were used to produce PAFC mixtures with superior impact resistance. The following conclusions were drawn from experimental research.

1. A reduced compressive strength was observed when natural aggregate was replaced for either a single or double layer of asphalt-coated aggregate. Compared to the natural aggregate specimens, the compressive strength of the single- and double-coated specimens without fibres was reduced by 5.08 and 11.02%, respectively. Similarly, 5.97 and 7.67% lower compressive strengths were found in the P-PF C1 and P-PF C2 specimens as compared to the P-PF specimen, while the compressive strength was reduced by 3.95 and 5.53% for P-SF-C1 and P-SF-C2 specimens, respectively, as compared to P-SF specimens.
2. Compared to NC, the compressive strength of the PF and SF-based specimens containing 100% natural aggregate increased by 25.17 and 48.06%, respectively. Fibrous specimens with single and double coatings showed a similar trend of strength enhancement. Fibres played a significant role in this development owing to their extraordinary ability to bridge micro and macro cracks.
3. Impact energies (EO1 and EO2) increased from a single layer of asphalt coating to a double layer. The EO1 and EO2 values of the single asphalt-coated aggregate specimens were 1.55 and 2.11 times higher than the natural aggregate specimens, respectively. The EO1 and EO2 values were 1.73 and 2.56 times greater for the double asphalt-coated specimens. An excellent post-cracking resistance was seen in the specimens when single- and double-layer asphalt-coated aggregates were employed.
4. The impact energy was improved by adding asphalt-coated aggregate into PAFC. The EO1 and EO2 values of the P-PF-C1 specimens were 1.85 and 2.91 times greater than those of NC-C1. These values were much greater in the P-SF-C1 specimens (by 2.65 and 8.79 times, respectively). There was a similar increase in strength in the double-coated aggregate fibrous concrete, which were 1.76 and 2.81 times greater EO1 and EO2 values for P-PF C2 specimens than NC-C2 specimens. For the P-SF-C2 specimens, the EO1 and EO2 values were 2.45 and 7.75 times higher than the NC-C2 specimen.
5. The post-cracking resistance of PAFC was improved significantly by adding fibres and marginally by adding asphalt-coated aggregate. The impact ductility index for the asphalt-coated aggregate improved from 1.2 to 1.8 for non-fibrous concrete, 2.4 to 2.9 for PF-based fibrous concrete and 5.3 to 5.8 for SF-based fibrous concrete. A brittle failure was observed in all non-fibrous specimens, while a ductile failure was observed in fibrous specimens irrespective of asphalt-coated aggregate usage.

## Figures and Tables

**Table 1:** Asphalt coating material properties.

Standard	Type of Test	Test Results	Recommended Value
IS 1206 (Part 3) [24]	Viscosity (Kinematic) at 135 °C (Cst)	487	400
IS 1206 (Part 2) [25]	Viscosity (Absolute) at 60 °C (poise)	3999	3200 to 4800
IS 1208 [26]	Ductility at 25 °C (cm)	48	25
IS 1206 (Part 1) [27]	Viscosity Ratio at 60° (Cst)	2.6	4
IS 1205 [28]	Softening point (°C)	50.6	50
IS 1203 [29]	Penetration at 25 °C,100 g, 5 s (mm)	36	35

**Table 2:** Mix combinations.

S. No	Mix Id	Cement/Sand Ratio	W/C	Fibre Type	Fibre Dosage * (%)	Fibre Kg/m^3^	Asphalt-Coated Aggregate (%)	Number of Asphalt Coating Layers	SP (%)
1	NC	1.0	0.42	-	0	0	0	0	0.3
2	NC-C1			-	0	0	100	1	0.3
3	NC-C2			-	0	0	100	2	0.3
4	P-PF			PF	3	235.5	0	0	0.45
5	P-PF-C1			PF	3	235.5	100	1	0.5
6	P-PF-C2			PF	3	235.5	100	2	0.5
7	P-SF			SF	3	235.5	0	0	0.45
8	P-SF-C1			SF	3	235.5	100	1	0.5
9	P-SF-C2			SF	3	235.5	100	2	0.5

* fibre dosage is 3% by volume of concrete.

**Table 3:** Results from impact test.

Mixture Id	Impact Numbers		Impact Energy (J)		Ductility Index
	O1	O2	EO1	EO2	
NC	22	27	447.8	549.5	1.2
NC-C1	34	57	692.0	1160.2	1.7
NC-C2	38	69	773.4	1404.4	1.8
P-PF	56	135	1139.8	2747.7	2.4
P-PF-C1	63	166	1282.3	3378.7	2.6
P-PF-C2	67	194	1363.7	3948.6	2.9
P-SF	86	460	1750.4	9362.7	5.3
P-SF-C1	90	501	1831.8	10,197.1	5.6
P-SF-C2	93	535	1892.9	10,889.2	5.8

## Data Availability

Not applicable.
